# Functional Characterization of Circadian Nuclear Receptors REV-ERBα and REV-ERBβ in Human Osteosarcoma Cell Cultures

**DOI:** 10.3390/ijms25020770

**Published:** 2024-01-07

**Authors:** Hana Cho, Ahee Yun, Joohee Kim, Eunjeong Park, Jong-Wha Jung, Sooyoung Chung, Gi Hoon Son

**Affiliations:** 1 Department of Biomedical Sciences, Korea University College of Medicine, Seoul 02841, Republic of Korea; prettyhana1@hanmail.net (H.C.); kim.jooheejudykim@gmail.com (J.K.); pinkcolony@korea.ac.kr (E.P.); 2 Department of Brain and Cognitive Sciences, Scranton College, Ewha Womans University, Seoul 03760, Republic of Korea; aheeyun@gmail.com; 3 Research Institute of Pharmaceutical Sciences, College of Pharmacy, Kyungpook National University, Daegu 41566, Republic of Korea; jungj@knu.ac.kr

**Keywords:** circadian clock, circadian rhythm, REV-ERBs, U2OS cell, transcriptome

## Abstract

REV-ERBα and its paralog, REV-ERBβ, encoded by *NR1D1* and *NR1D2* genes, are key nuclear receptors that link the circadian timing system and metabolic homeostasis. Since heme is an endogenous ligand, REV-ERBs have been considered key components of the circadian molecular clock and can be pharmacologically targeted to treat various circadian rhythm-related diseases, such as cardiometabolic, inflammatory, and neuropsychiatric diseases, as well as cancer. REV-ERBs are believed to be functionally redundant and compensatory, although they often affect the expression of gene subsets in an isoform-specific manner. Therefore, this study aimed to identify the redundant and distinct roles of each isoform in controlling its target genes by comparing the transcriptome profiles of a panel of mutant U2OS human osteosarcoma cells in which either *NR1D1* or *NR1D2* was ablated. Indeed, our transcriptomic analyses revealed that most REV-ERB-regulated genes are controlled by redundant or even additive actions. However, the RNA expression profiles of each single mutant cell line also provide strong evidence for isoform-dependent actions. For example, REV-ERBα is more responsible for regulating the NF-κΒ signaling pathway, whereas a group of extracellular matrix components requires REV-ERBβ to maintain their expression. We found that REV-ERBs have isoform-selective functions in the regulation of certain circadian output pathways despite their overlapping roles in the circadian molecular clock. Thus, the development of isoform-selective REV-ERB modulators can help treat metabolic disturbances and certain types of cancer.

## 1. Introduction

Circadian rhythms with an approximately 24 h period coordinate various biological processes. Transcription and translation feedback loops comprise a set of transcriptional regulators that underlie the hierarchically organized circadian timing system in mammals [1]. REV-ERBα and its paralog REV-ERBβ, which are encoded by nuclear receptor subfamily 1, group D, member 1 and 2 (*NR1D1* and *NR1D2*) genes, respectively, belong to the nuclear receptor superfamily of ligand-activated transcriptional regulators [2,3]. They serve as key transcriptional repressors of a stabilizing loop that constitutes the mammalian circadian molecular clock by competing with retinoic acid-related orphan receptors (RORs), thereby playing a pivotal role in the circadian control of ROR/REV-ERB-responsive element (RRE)-mediated transcriptional activities. The circadian mRNA expression expressed by the uppermost transcription factors (TFs) of the molecular clock, such as the circadian locomotor output cycles kaput (CLOCK) and brain and muscle aryl hydrocarbon receptor nuclear translocator-like protein 1 (BMAL1; also known as aryl hydrocarbon receptor nuclear translocator-like, ARNTL), are responsible for cyclic REV-ERB accumulation [4,5].

REV-ERBs are regarded as pharmacologically controllable components of the mammalian molecular clock owing to their important roles in orchestrating circadian physiology, metabolism, and behavior, as well as the molecular characteristics of nuclear receptors [6,7]. Since heme is an endogenous ligand that binds to REV-ERBs [8,9], several synthetic ligands that act on REV-ERBs have been developed [10,11,12,13,14,15,16]. However, little attention has been paid to identifying the functional and pharmacological differences between these two nuclear receptor isoforms, primarily because earlier studies have shown that REV-ERBs have redundant roles and can be compensated for by controlling the RRE-mediated rhythmic expression of canonical clock genes and circadian behaviors [4,5]. A cistrome-based comparison of two REV-ERB isoforms on murine chromatin showed that they shared more than 50% of binding sites [5]. Nevertheless, it can be conversely noted that REV-ERBα and β often recognize their binding sites in an isoform-selective manner. More importantly, several types of non-canonical modes of action have been reported. For example, REV-ERBs can compete with several nuclear receptors in cell-type and *cis*-element-dependent fashions [17,18].

Therefore, this study aimed to determine whether REV-ERBs have significant isoform-selective effects on the transcriptional regulation of their target genes at the genome-wide level. Accordingly, we prepared a panel of mutant U2OS cell lines bearing a functional deficiency of REV-ERB(s) using a CRISPR/Cas9-based gene truncation strategy. U2OS cells, part of a human osteosarcoma cell line, were used in this study because they are widely used to test the cyclic activity of human molecular clockworks in vitro [19].

## 2. Results

### 2.1. Mutant U2OS Cell Line Establishment

**Figure 1:**
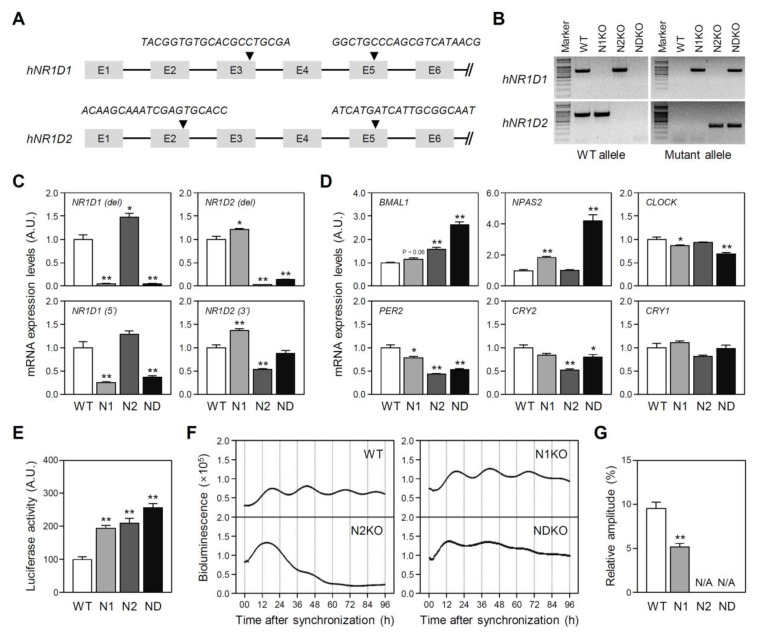
Generation of mutant U2OS cell lines with CRISPR/Cas9-mediated *NR1D1* and/or *NR1D2* gene deletions. (**A**) A schematic representation of sgRNA core sequences and their targeting sites on human *NR1D1* and *NR1D2* genes. (**B**) The PCR-based confirmation of mutant alleles (WT: wild-type U2OS cells; N1KO (N1): a mutant U2OS cell line with a truncated *NR1D1* gene; N2KO (N2): a mutant cell line with a truncated *NR1D2* gene; NDKO (ND): a mutant cell line with defective *NR1D1* and *NR1D2* genes). (**C**) *NR1D1* and *NR1D2* mRNA expression profiles in WT and mutant U2OS cells. *NR1D1* (*del*) and *NR1D2* (*del*) indicate the RT-qPCR results using primer sets specifically binding to the truncated regions (upper panels). Lower panels noted as *NR1D1* (5′) and *NR1D2* (3′) indicate the results using primer sets recognizing the remaining gene fragments. (**D**) mRNA expression profiles for canonical clock genes examined using RT-qPCR analyses. The relative mRNA expression levels in (**C**,**D**) are presented as the mean ± SEM in an arbitrary unit (A.U.), in which a mean expression level of the WT controls is set as 1 (n = 4 for each group; *: *p* < 0.05 and **: *p* < 0.01 by Student’s *t*-test). (**E**) Relative luciferase activities of the transfected m*Bmal1*-LUC reporter normalized by Renilla luciferase activities of co-transfected pRL-TK. Data are presented as the mean ± SEM of an A.U., in which a mean expression level of the WT controls is set as 1 (n = 4 for each group; *: *p* < 0.05 and **: *p* < 0.01 vs. WT cells using Student’s *t*-test). (**F**,**G**) Circadian properties of WT and mutant U2OS cell lines examined by dexamethasone (DEX)-synchronized m*Bmal1*-LUC reporter activities. (**F**) Representative plots showing detrended bioluminescence signals in the WT and mutant U2OS cell cultures. (**G**) Relative amplitude (AMP) of bioluminescence signals expressed as the mean ± SEM % of mesor in each plot (n = 4 for each group; **: *p* < 0.01 using Student’s *t*-test; N/A: not applicable).

To characterize REV-ERBα and/or REV-ERBβ-dependent gene expression, we generated a set of mutant human U2OS-based cell lines, in which CRISPR/Cas9-mediated gene deletion was evoked in *NR1D1* or *NR1D2* gene loci. The sgRNA sequences and their targeting sites are shown in Figure 1A. PCR-based genotyping with specific primer sets distinguishing wild-type (WT) and mutant alleles clearly showed *NR1D1* or *NR1D2* fragment deletion in each mutant cell line (Figure 1B). The *NR1D1*-mutant allele lacking a region between exons 3 and 5 was detected in *NR1D1*-truncated (N1KO) and *NR1D*-double-mutant (NDKO) cells but not in WT or *NR1D2*-truncated (N2KO) cells. In contrast, the *NR1D2*-mutant allele lacking the region between exons 2 and 5 was detected only in the N2KO and NDKO cell lines. RT-qPCR analyses confirmed gene deletion(s) in each mutant U2OS cell line (Figure 1C). A PCR using primer sets specifically recognizing truncated regions and barely detected *NR1D1* mRNA in N1KO and NDKO cells or *NR1D2* mRNA in N2KO and NDKO cells (Figure 1C, upper panels). Additional RT-qPCR experiments with primer sets specifically recognized the upstream (*NR1D1*) or downstream (*NR1D2*) of the truncated regions clearly demonstrated that truncated *NR1D* mRNA expression was reduced in each mutant cell line (Figure 1C, lower panels).

Next, we compared the steady-state canonical clock gene mRNA expression levels between the WT and *NR1D*-mutant U2OS cell lines (Figure 1D). *BMAL1* and *NPAS2* genes are under the repressive transcriptional control of REV-ERBs through the RRE elements located in their promoter regions [6,20]. In agreement with this notion, *BMAL1* and *NPAS2* mRNA expression levels in the NDKO cells were augmented by more than 2- and 4-fold, respectively, as well as to a lesser extent in N1KO or N2KO cells. Although *CLOCK* gene transcription is usually inhibited by REV-ERBs [21], *CLOCK* mRNA expression in U2OS cells was reduced by *NR1D1* gene abrogation. *PER2* and *CRY2* mRNA levels were also significantly lower in the mutant cells than those in the WT cells. In support of the increased *BMAL1* and *NPAS2* mRNA expression, normalized luciferase (LUC) reporter activity driven by the murine *Bmal1* promoter was significantly higher in all *NR1D*-mutant cell lines (Figure 1E). In contrast to the enhanced *Bmal1* promoter activity, cyclic promoter activation was attenuated in mutant cells, which were synchronized via acute dexamethasone (DEX) application. The relative amplitude of the rhythmic activity of the *Bmal1* promoter decreased in N1KO cells and was even abolished in N2KO and NDKO cell lines (Figure 1F,G). Collectively, these results indicate that the genetic ablation of *NR1D1* and/or *NR1D2* genes in the U2OS cells enhanced but dysregulated RRE-mediated gene transcription.

### 2.2. Differentially Expressed Gene Characterization in NDKO Cells

**Figure 2:**
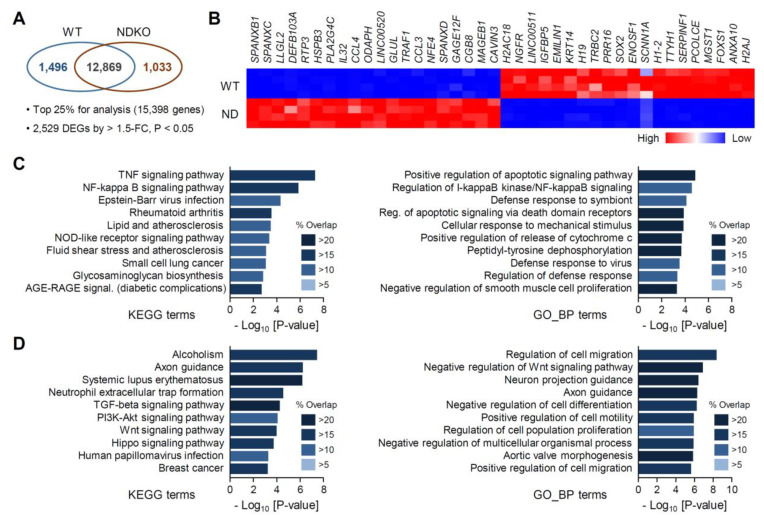
Differential gene expression profiles of *NR1D1/2* double mutant (NDKO/ND) cells in comparison with wild-type (WT) U2OS controls. (**A**) Venn diagram of the differentially expressed genes (DEGs) between WT and NDKO cells. Expression levels of 1496 and 1033 gene transcripts, among the 15,398 significantly expressed genes, were significantly higher in the WT and NDKO cells, respectively, as defined by >1.5-fold changes and *p* < 0.05 between the groups (n = 4 for each genotype). (**B**) A heat map representation for the top 20 upregulated and 20 downregulated DEGs of the NDKO cells. (**C**,**D**) Gene enrichment analyses for the (**C**) upregulated and (**D**) downregulated DEGs of the NDKO cells. The top significantly enriched terms were assessed using gene sets from the “KEGG Pathway” (left) or “GO Biological Process” (GO_BP, right) databases. Overlapping *p* values and % overlapping for the given KEGG/GO_BP terms are presented as color-coded bar charts.

We then compared the genome-wide RNA expression profiles of *NR1D*-mutant cell lines. Initially, we defined and characterized the differentially expressed genes (DEGs) in NDKO cells relative to those in the WT controls. Among the 15,398 significantly expressed gene transcripts, we identified 2529 DEGs (1033 upregulated and 1496 downregulated DEGs) between the two groups, using the criteria > 1.5-fold change (FC) and *p* < 0.05 (Figure 2A; Table S1 for the full list of DEGs). The top 40 DEGs in terms of fold differences (20 upregulated and 20 downregulated) are shown in Figure 2B; for example, *SPANXB1*, *SPANXC*, *LLGL2*, *DEFB103A*, and *RTP3* were the top five most upregulated genes for the abrogation of both *NR1D* genes, and *H2AJ*, *ANXA10*, *FOXS1*, *MGST1*, and *PCOLCE* were the most downregulated gene transcripts.

We then carried out the gene enrichment analyses of the DEGs to gain systemic insights into REV-ERB-regulated genes. Comparing the upregulated DEGs with multiple gene libraries such as the “Kyoto Encyclopedia of Genes and Genomes (KEGG) Pathway” and “Gene Ontology Biological Process (GO_BP)” using ENRICHR [22,23] suggested that genes constituting several biological pathways were significantly overrepresented among the NDKO DEGs (Figure 2C,D; see Table S2 for detailed information). For the upregulated gene transcripts, genes linked with the NF-κΒ signaling pathway (such as “TNF signaling pathway” and “NF-kappa B signaling pathway” among the KEGG terms and “Regulation of I-kappaB kinase/NF-kappaB signaling” among GO_BP terms) and several apoptosis-related terms were significantly enriched in the DEGs as supported by both KEGG and GO_BP gene lists (Figure 2C). On the contrary, genes controlling cellular motility/migration linked with cytoskeletal remodeling (such as “Axon guidance” in both gene libraries and “Regulation of cell migration” and “Positive regulation of cell motility” among GO_BP terms) as well as the Wnt signaling pathway were enriched among the downregulated DEGs of the NDKO cells (Figure 2D). Our findings imply that these signaling pathways may be controlled by the stabilizing loop of the circadian molecular clock, at least in certain cell types.

**Figure 3:**
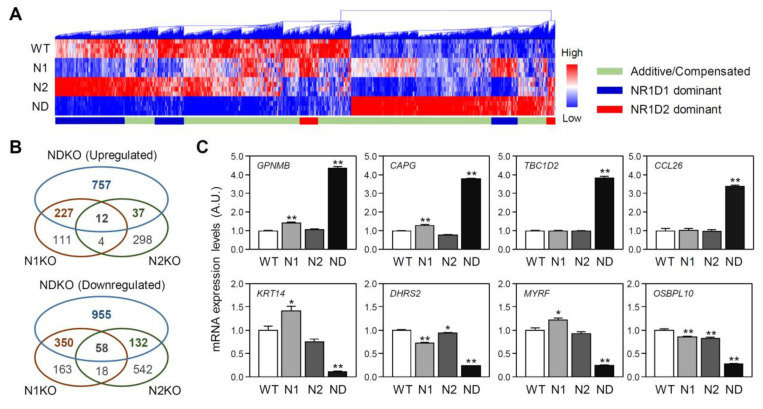
Comparison of differentially expressed genes (DEGs) relative to the WT U2OS cells among *NR1D*-mutated cell lines. (**A**) A heat map representation with hierarchical clustering of the DEGs obtained from the *NR1D1/2* double mutant (NDKO) cells for all tested cell lines. Gene clusters were divided into three categories, as indicated, according to RNA expression patterns in *NR1D1* and *NR1D2* mutant cells (N1KO and N2KO, respectively). (**B**) Venn diagrams comparing the DEGs among *NR1D*-mutated cells. Significantly upregulated (upper) or downregulated (lower) genes from each mutant cell line, as defined by >1.5-fold changes and *p* < 0.05 in comparison with the WT cells, are separately shown. (**C**) DEGs with distinctively larger fold changes in the NDKO cells (ND) than those found in the N1KO (N1) or N2KO (N2) cells. mRNA expression levels for a given RNA species were calculated from the transcripts per million (TPM) values of individual cells and expressed as the mean ± SEM of an arbitrary unit (A.U.), in which the mean expression level of the WT controls is set as 1 (n = 4 for each group; *: *p* < 0.05 and **: *p* < 0.01 vs. WT cells using Student’s *t*-test).

Notably, more than 67% of NDKO DEGs exhibited significant alterations only in these double mutant cells or more robust fold changes in NDKO cells than in N1KO or N2KO cells (Figure 3A,B). Indeed, 73.28% (757/1033 genes) of upregulated genes and 63.88% (955/1495 genes) of downregulated genes were significantly altered only in the NDKO cells but not in the N1KO or N2KO cells (Figure 3B). For example, *GPNMB*, *CAPG*, *TBC1D2*, and *CCL26* mRNA expression levels were increased by more than 3- or 4-fold in NDKO cells. Several downregulated DEGs, such as *KRT14*, *DHRS2*, *MYRF*, and *OSBPL10*, exhibited drastic reductions in their expression in NDKO cells (Figure 3C). These features can be attributed to the additive actions of both REV-ERBα and β or a compensatory mechanism between them. This notion is in good accordance with previous reports, which demonstrated the redundant functions of REV-ERBα and β in controlling behavioral rhythms in mice and cyclic *Bmal1* gene transcription in cultured fibroblasts [4,5].

### 2.3. Isoform-Specific Target Genes among the NDKO DEGs

**Figure 4:**
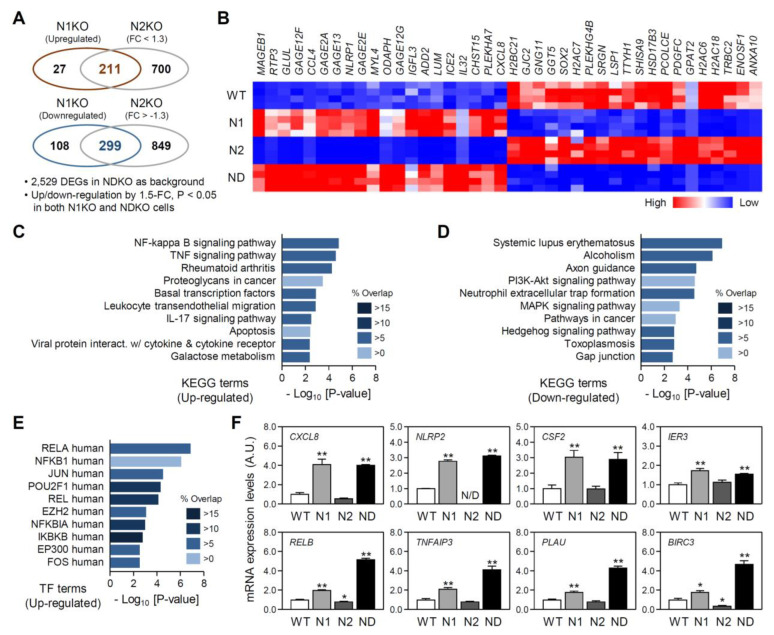
Characterization of REV-ERBα-regulated differentially expressed genes (DEGs). (**A**) Venn diagrams showing the number of RNA species significantly upregulated or downregulated in both *NR1D1*-mutant (N1KO) and *NR1D1/2*-double-mutant (NDKO) cells but showing modest changes (<1.3-fold) in *NR1D2*-mutant (N2KO) cells. (**B**) A heat map representation for the top 20 upregulated and 20 downregulated genes identified as the REV-ERBα-regulated NDKO DEGs. (**C**,**D**) Pathway enrichment analyses for the REV-ERBα-dependently upregulated (**C**) and downregulated NDKO DEGs (**D**). The top significantly enriched terms were assessed using gene sets from the “KEGG Pathway” database. Overlapping *p* values and % overlapping for the given pathway term are summarized as color-coded bar charts. (**E**) Top 10 upstream transcriptional factors (TFs) enriched for the upregulated REV-ERBα-regulated NDKO DEGs, as suggested by the “TRRUST TFs” gene list in the ENRICHR. Overlapping *p* values and % overlapping for the given transcriptional regulator are presented as a color-coded bar chart. (**F**) Relative expression profiles for the representative DEGs constituting the NF-κΒ signaling pathway or suggested as transcriptional target genes of the NF-κΒ complex. mRNA expression levels for a given RNA species were calculated from the transcripts per million (TPM) values of individual cells and expressed as the mean ± SEM of an arbitrary unit (A.U.), in which a mean expression level of wild-type (WT) controls is set as 1 (n = 4 for each group; *: *p* < 0.05 and **: *p* < 0.01 vs. WT cells using Student’s *t*-test).

Although most REV-ERB-regulated genes were more robustly or even dominantly affected by the ablation of both REV-ERBs, a significant portion of the DEGs exhibited biased regulatory profiles upon the deletion of *NR1D1* or *NR1D2* (Figure 3B). Therefore, we focused on NDKO DEGs predominantly affected by one of the two isoforms. Accordingly, we defined NDKO DEGs that were significantly upregulated in both N1KO and NDKO cells, but with a <1.3-fold increase in the N2KO cells, as the REV-ERBα-upregulated genes (211 DEGs; Figure 4A, upper), and those significantly downregulated in the N1KO and NDKO cells, but with a >1.3-fold change in the N2KO cells, as the REV-ERBα-downregulated genes (299 genes; Figure 4A, lower). The top 40 DEGs in terms of fold difference (20 upregulated and 20 downregulated) are shown in Figure 4B. *MAGEB1*, *RTP3*, *GLUL*, *GAGE12F*, and *CCL4* were the top five REV-ERBα-upregulated genes, and *H2AC6*, *H2AC18*, *TRBC2*, *ENOSF1*, and *ANXA10* were the most REV-ERBα-downregulated genes.

Interestingly, gene enrichment analyses revealed that key gene transcripts constituting certain pathways and biological processes overrepresented by the NDKO DEGs are significantly enriched in REV-ERBα-dominant DEGs (Figure 4C,D; see Table S3 for more detailed information). For example, KEGG pathway terms associated with NF-κΒ- or cytokine-related pathways such as “NF-kappa B signaling pathway”, “TNF signaling pathway”, “IL-17 signaling pathway”, and “Viral protein interaction with cytokine & cytokine receptor” are among the top 10 pathway terms enriched by REV-ERBα-upregulated genes (Figure 4C). Enriched KEGG terms such as “Systemic lupus erythematosus”, “Alcoholism”, “Axon guidance”, and “PI3K-Akt signaling pathway”, ranked as overrepresented terms by the downregulated NDKO DEGs, were mainly affected by REV-ERBα abrogation (Figure 4D). In accordance with enriched KEGG terms, upstream TF enrichment analyses using “TRRUST TFs” gene libraries suggested that the abrogation of REV-ERBα impacts mRNA expression profiles for a set of genes consisting of NF-κΒ signaling pathway to subsequently affect the transactivation of target promoters using the NF-κΒ complex. Indeed, half of the top 10 enriched TF terms predicted via REV-ERBα-dominantly upregulated genes were transcriptional regulators mediating the NF-κΒ signaling pathway (Figure 4E). The RNA expression profiles of representative genes constitute and/or are regulated by the human NF-κΒ signaling pathway (Figure 4F). For example, *CXCL8*, *NLRP2*, *CSF2*, and *IER3* mRNA expression profiles were exclusively affected by REV-ERBα. Several genes, such as *RELB*, *TNFAIP3*, *PLAU*, and *BIRC3*, were apparently under the suppressive influence of REV-ERBα, although ablation of both REV-ERB isoforms further augmented their mRNA expression levels.

**Figure 5:**
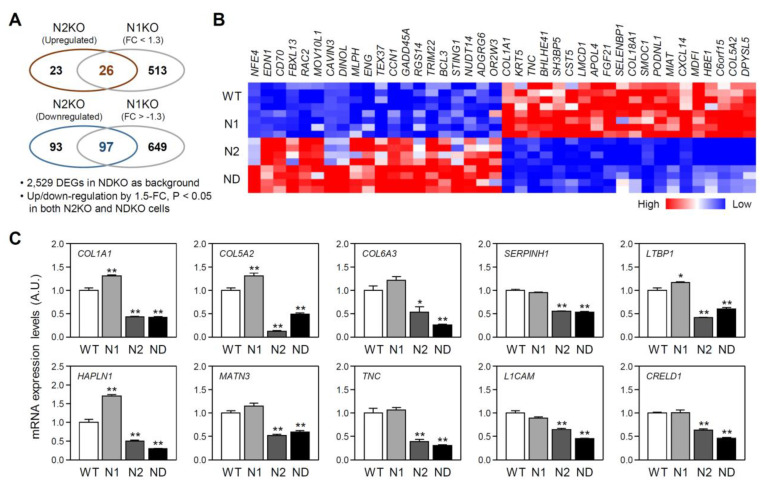
Characterization of REV-ERBβ-regulated differentially expressed genes (DEGs). (**A**) Venn diagrams show the number of gene transcripts significantly upregulated or downregulated in both *NR1D2*-mutant (N2KO) and *NR1D1/2*-double-mutant (NDKO) cells but showing modest changes (<1.3-fold) in *NR1D1*-mutant (N1KO) cells. (**B**) A heat map representation for the top 20 upregulated and 20 downregulated genes identified as REV-ERBβ-regulated NDKO DEGs. (**C**) Relative expression profiles for the REV-ERBβ-regulated DEGs coding for a subset of proteins constituting the extracellular matrix (ECM). mRNA expression levels for a given RNA species were calculated from the values of transcripts per million (TPM) of individual cells and expressed as the mean ± SEM of an arbitrary unit (A.U.), in which a mean expression level of wild-type (WT) controls was set as 1 (n = 4 for each group; *: *p* < 0.05 and **: *p* < 0.01 vs. WT cells using Student’s *t*-test).

In contrast, the mRNA expression profiles of some NDKO DEGs were influenced more by REV-ERBβ. For the same criteria as those of REV-ERBα, we defined a set of REV-ERBβ-regulated genes (Figure 5A; 26 DEGs for REV-ERBβ-dependent upregulation and 97 for downregulation). The top 40 REV-ERBβ-regulated DEGs (20 upregulated and 20 downregulated) are shown in Figure 5B; *NFE4*, *EDN1*, *CD70*, *FBXL13*, and *RAC2* were the top 5 REV-ERBβ-dominantly upregulated genes, and *MDFI*, *HBE1*, *C6orf15*, *COL5A2*, and *DPYSL5* were the most REV-ERBβ-downregulated genes. Although our gene enrichment analyses using KEGG gene libraries did not present significantly overrepresented pathway terms, genes constituting the extracellular matrix (ECM) were highly enriched among REV-ERBβ-regulated genes, as suggested by their comparison with the “GO Cellular Component” (GO_CC; see Table S4 for more detailed information) gene library. Among ECM protein-coding DEGs, *COL6A3*, *SERPINH1*, *MATN3*, *TNC*, *L1CAM*, and *CRELD1* mRNA expression levels were significantly downregulated in both N2KO and NDKO cells but unchanged in N1KO cells. Moreover, the mRNA levels of several ECM-related gene transcripts, such as *COL1A1*, *COL5A2*, *LTBP1*, and *HAPLN1*, were augmented in N1KO cells compared to those in WT cells but were significantly reduced in both mutant cell lines lacking functional REV-ERBβ expression regardless of REV-ERBα (Figure 5C). Taken together, despite their redundant roles in controlling the circadian clock, our transcriptome analyses strongly suggest the presence of the isoform-specific actions of REV-ERBs, at least in human U2OS cells.

## 3. Discussion

This study established a panel of mutant U2OS cell lines lacking either the REV-ERBα or REV-ERBβ expression using CRISPR/Cas9 and a dual sgRNA-mediated gene deletion strategy to identify the redundant and isoform-specific roles of these circadian nuclear receptors. Our transcriptomic analyses of the mutant cell lines revealed that a majority of REV-ERB-regulated genes were controlled by redundant or even additive actions. However, the RNA expression profiles of each single mutant cell line provided strong evidence for the presence of isoform-dependent actions in human osteosarcoma cell lines. As REV-ERBs lack the C-terminal activation domain, unlike other canonical nuclear receptors, they primarily repress the transcription of target genes upon their monomeric or dimeric binding to the RRE motifs of *cis*-element consisting of one or repeated AGGTCA nuclear receptor half-sites along with 5′ A/T-rich flanking sequence [24,25]. Both REV-ERBα and β recognize the canonical *cis*-element(s) and recruit the nuclear receptor corepressor (NCoR) complex to inhibit the transactivation of target promoters [26].

Previous studies have demonstrated the redundant functions of REV-ERBα and β in the periodic regulation of circadian phenotypes both in vivo and in vitro [4,5,27], but isoform-specific actions have also been implicated, particularly in regulating metabolic and physiological functions. For example, REV-ERBα and β may have distinct roles in controlling metabolic functions in skeletal muscle cells. The comparison of REV-ERBα and β knock-out (KO) mice showed their opposite roles in controlling the muscular expression of mitochondrial and fatty acid oxidation genes [28]. In addition, the isoform-specific anti-proliferative or cytoprotective functions of REV-ERBα and β have been proposed in certain cancer cells [3,29]. These features can be attributed to the cell type-dependent differential expression of REV-ERB isoforms [29]. However, it should be noted that *NR1D1* and *NR1D2* mRNA expression levels in U2OS cells are comparable (Table S1). More importantly, approximately 67% of NDKO DEGs are consequences of the abrogation of both isoforms, indicating that both REV-ERBs are functional and even compensate for each other in U2OS cells. In this context, it is noteworthy that REV-ERBs permit wide variability in *cis*-elements and often bind to genomic sites lacking the canonical RRE motif tissue specifically. Lazar and colleagues demonstrated that REV-ERB-binding sites depend highly on the cell type and imply several interactions with other TFs; chromatin immunoprecipitation sequencing (ChIP-seq) analyses revealed that REV-ERBα or β could occupy canonical RRE motifs as well as multiple *cis*-elements recognized by other classes of transcriptional regulators such as HNF4A, HNF6, NF1/FOXA1 and CEBPA [5,18].

Although REV-ERBs are primarily considered transcriptional repressors, several genes were significantly downregulated in the absence of functional REV-ERBs (see Figure 2A). Similar to our findings, a significant portion of differentially expressed hepatic gene transcripts exhibited lower expression levels in REV-ERBα KO mice than in the WT controls but were augmented in transgenic mice, which overexpressed hepatic REV-ERBα throughout the day [30]. Genes encoding transcriptional repressors under the suppressive control of REV-ERBs, such as the nuclear factor, interleukin 3-regulated (NFIL3), and a transcriptional repressor belonging to the D-box family, may account for REV-ERB-evoked transcriptional activation [31]. On the contrary, REV-ERBα often cooperates with other transcriptional regulators to induce the transcriptional activation of target genes upon binding to the promoter region. For example, SP1, a zinc finger TF binding to GC-rich motifs, forms a complex with REV-ERBα, but not with REV-ERBβ, to activate the transcription of the *Gja1* gene encoding connexin 43 [32,33]. REV-ERBα also activates the *Ddit3* gene (also known as the CEBP homologous protein) transcription in mouse hepatoma cells, and the REV-ERB-induced gene transcription is functionally inhibited by small heterodimer partner (SHP) nuclear receptor RRE independently [34]. Along with the differential occupations of non-RRE motifs by REV-ERBα and β, as noted earlier [5], these findings strongly suggest that REV-ERBα and β have isoform-specific roles in the indirect or non-canonical control of target gene expression, which involve protein–protein interactions with other TFs.

In conclusion, REV-ERBα and β are largely redundant and compensate each other to control target gene expression. Nevertheless, they also affect the RNA expression of a subset of gene isoforms specifically, even in the same cell type, supporting their distinct roles. In particular, REV-ERBα affects the NF-κΒ pathway, which mediates a wide spectrum of cytokine/chemokine signaling. On the contrary, REV-ERBβ maintains the expression of a group of ECM components. Considering the multimodal actions of REV-ERBs in terms of their functional interactions with a wide range of other TFs [5,18,31,32,33,34], it is reasonable that isoform-specific REV-ERB-regulated genes may be dependently manifested by cell type in association with the cellular contexts of transcriptional regulators. Since heme is an endogenous ligand [8,9], REV-ERBs have been considered a key component of the mammalian circadian clock, which serves as the primary molecular target of several lines of small molecules [2,3]. Although the distinct roles of each REV-ERB isoform in the regulation of circadian output pathways have been implicated, the synthetic REV-ERB ligands developed thus far have not yet focused on isoform selectivity. Therefore, the development of isoform-selective REV-ERB modulators could be beneficial, particularly for treating metabolic disturbances and certain types of cancer.

## 4. Materials and Methods

### 4.1. Plasmids

Plasmids for the simultaneous expression of human codon-optimized Cas9 and the indicated sgRNAs were prepared using a commercial vector construction service (Vector Builder Inc., Chicago, IL, USA). A pair of sgRNAs for a target gene was ligated into a dual sgRNA expression plasmid (VectorBuilder). The CRISPR-targeted sequences are as follows: *hNR1D1* sgRNA #1: 5′-GGC TGC CCA GCG TCA TAA CG-3′; *hNR1D1* sgRNA #2: 5′-TAC GGT GTG CAC GCC TGC GA-3′; *hNR1D2* sgRNA #1: 5′-ATC ATG ATC ATT GCG GCA AT-3′; *hNR1D2* sgRNA #2: 5′-ACA AGC AAA TCG AGT GCA CC-3′. Mouse *Bmal1* promoter-driven firefly luciferase (mBmal1-LUC) and thymidine kinase promoter-driven Renilla luciferase (pRL-TK) reporters were used for bioluminescence recording, as described previously [17].

### 4.2. Cell Cultures and Generation of Mutant Cell Lines

U2OS (ATCC HTB-96) cells were mainly cultivated in Dulbecco’s modified Eagle’s medium (DMEM; Thermo Fisher Scientific, Waltham, MA, USA) supplemented with 10% fetal bovine serum (FBS; Thermo Fisher Scientific) and 1% of an antibiotic–antimycotic solution (Thermo Fisher Scientific) in a humidified incubator containing 5% CO_2_ at 37 °C. To generate N1KO or N2KO cell lines, U2OS cells were seeded onto a 6-well culture plate (SPL Life Sciences, Pocheon, Republic of Korea) and transfected with 2 μg of the sgRNA expression vector using the Lipofectamin 3000 Transfection Reagent (Thermo Fisher Scientific). After transfecting for 48 h, the cells were selected in the presence of 2 μg/mL of puromycin (Sigma-Aldrich, St. Louis, MO, USA). Cellular colonies were individually transferred to 24-well culture plates (SPL Life Sciences) and further cultured for expansion. NDKO cell lines were produced via sequential sgRNA-expressing plasmid transfection. Aliquots from each cell line were genotyped. Genomic DNA was extracted from WT and mutant U2OS cells using the LaboPass Tissue Genomic DNA Isolation Kit (Cosmo Genetech Inc., Seoul, Republic of Korea). Gene truncation at the intended genomic regions was confirmed using a PCR-based genotyping method, as described previously [35]. The primer sequences for genotyping are as follows: *hNR1D1* genotyping WT forward: 5′-TCT CCT GCT CAC CTG CT-3′; *hNR1D1* genotyping WT reverse: 5′-ACA GGA TGA GAA CAG CAT CAG-3′; *hNR1D1* genotyping KO forward: 5′-CTT TTC CCT CCC TGG ATC TC-3′; *hNR1D1* genotyping KO reverse: 5′-CAT GGA GAA ATC CTC CCA GA-3′; *hNR1D2* genotyping forward (common): 5′-TCT TCT GTT CCA TCT TCT CCA AAT A-3′; *hNR1D2* genotyping WT reverse: 5′-CCA GAT ATG CAA CCA GAA CTA AGA-3′; *hNR1D2* genotyping KO reverse: 5′-GGT AAC TAT TCT TGT TCT CAT TCT G-3′.

### 4.3. Total RNA Sample Preparation and Reverse Transcription-Quantitative PCR (RT–qPCR)

WT or mutant U2OS cells were plated in 6-well culture plates (SPL Life Sciences), further cultivated for 48 h, and then subjected to RNA isolation. The RT-qPCR was performed as previously described [17]. Briefly, the total RNA was isolated using the microRNeasy Mini Kit according to the manufacturer’s protocol (Qiagen, Hilden, Germany), and 1 μg of the RNA sample was then reverse-transcribed using the PrimeScript™ 1st Strand cDNA Synthesis Kit (Takara Bio, Kusatsu, Japan). The synthesized cDNA aliquots were subjected to qPCR using the Power SYBR Green PCR Master Mix (Thermo Fisher Scientific). Relative mRNA expression levels of the indicated canonical clock genes were calculated using the comparative Ct method, and the TATA-binding protein (*TBP*) mRNA was used as an internal control. The primer sequences used for RT-qPCR are listed in Supplementary Table S5.

### 4.4. Real-Time Bioluminescence Monitoring and Luciferase Reporter Assay

For real-time bioluminescence monitoring, WT or mutant U2OS cells were plated in 35 mm dishes (SPL Life Sciences) and transfected with a mixture of mBmal1-LUC (800 ng/well) and pRL-TK (200 ng/well) reporter plasmids for 24 h. After recovery, the cells were synchronized with 200 nM DEX for 2 h. The medium was then replaced with a recording medium containing 0.1 mM of D-luciferin (Promega, Madison, WI, USA). Light emission for 2 min at intervals of 20 min was integrated using a dish-type wheeled luminometer (Kronos-Dio, ATTO Cooperation, Tokyo, Japan). Background-subtracted bioluminescence profiles were analyzed using Cosinor analysis software (available at http://www.circadian.org, accessed on 7 September 2023). After more than 4 days of bioluminescence monitoring, cell extracts were prepared by incubating them in a 0.3 mL reporter lysis buffer for 15 min at room temperature. Luciferase activity was measured using a commercial Dual-Luciferase Reporter Assay Kit (Promega).

### 4.5. RNA Sequencing (RNA-Seq) and Gene Enrichment Analyses

The integrity of each total RNA sample was evaluated using an Agilent 2100 Bioanalyzer (Agilent Technologies, Santa Clara, CA, USA). RNA-seq and subsequent gene enrichment analyses were performed as previously described with minor modifications [36]. Briefly, poly-A RNA isolation from total RNA and cDNA library preparation was carried out using the Illumina Stranded mRNA Prep Kit (Illumina, San Diego, CA, USA) according to the manufacturer’s instructions. Primary sequence data were acquired using paired-end sequencing on a NovaSeq 6000 platform (Illumina), and the trimmed sequence reads were mapped to the human reference genome (Ensembl GRCh38). Normalized gene-level read counts in transcripts per million (TPM) were used to evaluate the gene expression profiles. Genes with an upper 25% expression level (15,398 genes) were considered as significantly detected transcripts, and DEGs at >1.5-FC and *p* < 0.05 (n = 4 for each genotype) were used to construct a primary dataset. We utilized ENRICHR, a web-based tool for gene enrichment analyses on selected lists of genes [22,23] (https://maayanlab.cloud/Enrichr/; accessed on 7 September 2023) and mainly used gene set libraries to explore gene enrichment as follows: the “Kyoto Encyclopedia of Genes and Genomes (KEGG) Pathway” and “Gene Ontology (GO) Biological Process” to explore biological pathway enrichment, “Gene Ontology (GO) Cellular Component” to categorize the subcellular locations of the DEGs, and “TRRUST TFs” to predict putative upstream the transcriptional regulators of the DEGs.

### 4.6. Statistics

Student’s *t*-test was used for simple comparisons between WT and mutant cell lines. For gene enrichment analysis, Fisher’s exact test was used to statistically evaluate the significance of overlaps between the input DEG list and the gene sets of the indicated library. Differences between groups were considered statistically significant at *p* < 0.05.

## 5. Conclusions

REV-ERBs have isoform-dependent functions in the regulation of certain circadian output pathways despite their overlapping roles in controlling the overt circadian rhythms. Thus, the development of isoform-selective REV-ERB modulators could be beneficial, particularly for treating metabolic disturbances and certain types of cancer.

## Data Availability

The raw RNA-seq data are available from the Gene Expression Omnibus (GEO) Repository (accession number: GSE248721).

## Supplementary Materials

The following supporting information can be downloaded at: https://www.mdpi.com/article/10.3390/ijms25020770/s1.
